# Characteristics of infant deaths with positive hair toxicology

**DOI:** 10.1007/s12024-026-01174-0

**Published:** 2026-01-26

**Authors:** N. Thomson, J. Fronczek, M. Lynch, J. L. Schumann

**Affiliations:** 1 Department of Forensic Medicine, Monash University, Southbank, VIC Australia; 2 Victorian Institute of Forensic Medicine, Southbank, 65 Kavanagh Street, VIC 3006 Australia; 3 Monash Addiction Research Centre, Frankston, VIC Australia

**Keywords:** Sudden infant death syndrome, Hair toxicology, Pathology, Infant death, Drug exposure

## Abstract

Sudden Unexpected Death in Infancy (SUDI) is the term given to all unexpected infant deaths, comprising accidental, non-accidental and natural deaths including Sudden Infant Death Syndrome (SIDS). As in other developed countries, Australian SUDI rates are relatively low, but incidence rates have remained essentially unchanged since 2004. To increase our understanding of these deaths, we reviewed a cohort of SUDI cases with positive hair toxicology, focussing on toxicology results, pathology findings, and SIDS risk factors. All infant deaths between 1*st* January 2014 and 31*st* of December 2023 with positive hair toxicology in the state of Victoria, Australia, were reviewed for toxicology results, pathology findings and SIDS risk factors. A total of 99 infant deaths met the selection criteria. At least one SIDS risk factor was reported in every case, with a median of 5 per case, highlighting that these infants likely had a high cumulative risk for SIDS. Co-sleeping was most common, reported in over 75% of cases. Polysubstance use was high (87%), with a median of 4 drugs detected per case. Methylamphetamine was detected in hair in over half of cases (*n* = 57, 58%). Postmortem findings including serious illnesses, like pneumonia (*p* = 0.002), significant neuropathology (*p* = 0.002 and *p* = 0.035), low head circumference (< 15th percentile) (*p* = 0.035) and low body weight (< 15th percentile) (*p* = 0.022 and *p* = 0.011) were all positively correlated with drug exposure in hair. Infants with positive hair toxicology appear to have a high cumulative risk for SIDS, which may reflect a chaotic home environment. Most postmortem findings were positively correlated with drug exposure.

## Key points

• Infants in Victoria who die with positive hair toxicology were exposed to multiple simultaneous risk factors for SIDS (median of 5 per case).

• Polysubstance exposure was common in these cases (87%) with a median of 4 drugs per case.

• There was a significant correlation (*p* < 0.05) between most postmortem findings examined and the presence of one or more drugs.

• A more comprehensive understanding of the prevalence and impact of drug exposure in cases of infant death may provide a viable avenue to reduce infant mortality in Australia.

## Introduction

Infant mortality, defined as the death of a child under the age of 1, has significantly decreased over recent decades but remains a serious global health issue [1]. The World Health Organisation (WHO) reports that Australia has a relatively low infant mortality rate of 3.1 per 1000, compared to the global average of 27.9 per 1000 [2]. However, it remains a significant concern in Australia, comprising almost 70% of all deaths among children [3]. Despite rates comparable with other developed countries, Australia has seen little decrease in the rate of Sudden Unexpected Death in Infancy (SUDI) since 2004 [4].

A recent study from the United States highlighted that the cause of death is not always clear, remaining unascertained in approximately a third (32%) of infant death cases [5]. SUDI includes all unexpected infant deaths with a broad range of causes including drowning, injury, unknown genetic conditions and Sudden Infant Death Syndrome (SIDS) [6]. SIDS form a subset of SUDI cases involving the sudden death of an infant during sleep, with the cause remaining unexplained following a full forensic investigation [7].

The *Triple Risk Model* remains one of the most widely accepted models for SIDS risk worldwide, acknowledging that while SIDS is not exclusive to infants with risk factors, cumulative risk is a significant danger, with the risk of SIDS directly proportionate to the number of risk factors present [8]. Filiano and Kinney defined SIDS risk factors as being either intrinsic (an underlying physiological vulnerability) or extrinsic (environmental factors including sleep position or co-sleeping) [9, 10]. Parental drug use is unique in that it can contribute to both intrinsic and extrinsic risk: maternal drug use during pregnancy is associated with both preterm birth and low birth weight [11], while pre or postnatal exposure to certain drugs can impact arousal levels and autonomic activity, preventing the infant from mounting an appropriate and protective response to an environmental stressor [9, 12]. Drug impairment by parents may also present as an extrinsic risk, manifesting as neglect, or a chaotic home environment with a dangerous sleep environment for the infant [13, 14].

Given the unexpected and unexplained nature of SUDI, these cases are typically investigated by a coroner or medical examiner [15]. However, SIDS is a diagnosis of exclusion, meaning there are no pathognomonic markers for its identification at autopsy [16]. Deaths are classified into four categories (SIDS IA, SIDS IB, SIDS II and Unclassified Sudden Infant Death), dependent on the tests performed, autopsy findings and the risk factors noted at time of death [6]. Toxicological analysis may be critical in determining the cause of death if drug-related, using specimens including blood, urine and hair [17]. Blood and urine are considered standard toxicological samples, detecting drug administration from hours or days previous, depending on the drug [18]. However, blood and urine analysis does not allow for an investigation of chronic or repetitive drug exposure in one test, nor does it detect remote environmental exposure alongside direct administration [19]. Toxicological analysis of hair in deceased infants is an increasingly valuable tool which can establish a history of chronic drug exposure in the time leading up to the death [18]. Infants typically have finer, more porous and less pigmented hair than adults, allowing for the detection of environmental drug exposure [20].

While drug exposure in children has been investigated, studies often focus on technical aspects of infant hair toxicology or explore toxicology findings in a wider age range outside of SUDI [21–23]. Less is known regarding the correlation of toxicology and pathology findings in SUDI cases in the context of positive hair toxicology. This is particularly important given the recognised difficulties in interpreting these findings in isolation [18]. The presence of drugs in hair and the pathology findings in cases of infant death need to be interpreted in conjunction with each other when determining cause of death. This study therefore aimed to examine a series of infant death cases with positive hair toxicology to determine if there is a correlation between drug exposure and adverse postmortem findings, while documenting risk factors for SIDS present.

## Methodology

All cases in this study involved coronial data collection from the Victorian Institute of Forensic Medicine (VIFM) internal Case Management System (iCMS). VIFM is the statutory authority for forensic medicine in Victoria, assisting in around 7000 coronial cases each year [23, 24]. On admission to VIFM, toxicological specimens, including blood, urine and hair, are collected and promptly analysed. In cases involving hospitalisation, ante-mortem toxicology specimens are also obtained when possible. For toxicological analysis, validated Liquid Chromatography-Tandem Mass Spectrometry (LC-MS/MS) methods are used to detect a wide range of commonly used pharmaceutical and illicit drugs [25]. Currently, Victoria is the only state in Australia which routinely performs hair toxicology following an infant death. Prior to autopsy, a full body CT scan and skeletal survey are performed [26]. At VIFM, an autopsy involves both an external and internal examination of major body systems and organs, both macroscopically and histologically. A range of ancillary tests are also performed, including microbiology and biochemistry. These findings are compiled to form the autopsy report. In cases where the next of kin object to a full internal autopsy, only an external examination is performed, the findings of which are reported in an inspection report.

### Study design and case collection

This study involved a retrospective descriptive case series design, examining forensic and medico-legal data. Following investigation, a collection of reports relating to the death, including autopsy findings, the toxicology report, the coroners finding, and a police summary of circumstances, are available on the iCMS for approved users. A Structured Query Language (SQL) protocol was used to source all infant deaths ($\:\le\:$12 months age) with positive hair toxicology between 1 st January 2014 and 31 st December 2023. This extraction included age, sex, cause of death and toxicology results. Cases were included regardless of whether a full autopsy or only external examination was performed. Information examined in further detail included pathology and toxicology findings, circumstances of the case and potential risk factors using the autopsy and toxicology reports, police summary of circumstances, and the SUDI checklist, which is a form allowing for the collection of information by police surrounding common SIDS risk factors including co-sleeping and sleep position. The Department of Human Services (DHS) response form was also examined, containing information on possible contact of the child with Child Protective Services (CPS). Eleven different SIDS risk factors were collected, based on the information available from the SUDI checklist. Significant pathology findings namely serious illness, congenital abnormalities and serious neuropathology findings were noted as either present or absent for each case. To explore growth and development, the markers of body weight and head circumference were used, and converted into percentiles based on the WHO standards (50th percentile is average for age and sex) [27].

### Data analysis

The eleven risk factors collected from iCMS for each case were transferred into Microsoft Excel and coded for analysis. Given the number of cases for some drugs was low, only drugs that were detected in more than 10 cases were analysed further; this allowed for larger categories to increase statistical power. Drugs exclusively administered in a hospital setting (e.g. midazolam) were excluded from analysis. Although drugs including morphine, codeine and amphetamine can be primary drugs or metabolites of other drugs, they were documented as separate drugs, with specific codetections that may reflect metabolites noted (e.g., morphine with codeine). These results were interpreted in collaboration with a forensic toxicologist. For pathological analysis, head circumference and body weight were classified into their WHO percentiles and congenital abnormalities noted as present or absent. While neuropathology findings and illnesses were common, some of these findings are considered incidental and common among children this age. Accordingly, this data was interpreted by forensic pathologists, with findings classified as serious illness (causing significant symptoms or requiring medical attention); serious neuropathology; or incidental findings (mild inflammation or birth trauma), the latter were excluded from analysis.

### Statistical analysis

Data were analysed using SPSS (Version 29). Descriptive statistics were generated as frequencies and percentages for categorical variables, or as medians and interquartile ranges (IQRs) for continuous variables. Descriptive statistics were used to estimate the prevalence for each risk factor, as well as the number and frequency of drugs detected in each case. Cases missing one or more factors were still included in analysis. Fisher’s exact test was used to correlate toxicology and pathology results and determine significance (p = < 0.05).

### Ethics

This project was approved by the VIFM Ethics Committee (ID: 1293).

## Results

### Risk factor analysis

A total of 99 cases met the inclusion criteria for this study and were included in analysis, most of which had a full autopsy performed (*n* = 84, 85%). Males accounted for approximately two thirds of cases (*n* = 62, 62%) while the median age at time of death was nine weeks old (range: 0–49 weeks) (Table 1). At least one risk factor was present in all cases, with a median of five risk factors per case (range: 1–8). This included risk factors such as smoke exposure (*n* = 52, 80%) and dangerous sleep position (*n* = 25, 36%). Co-sleeping was common, present in over three quarters of cases (*n* = 59, 78%). While illicit drugs were present in almost three quarters of cases (*n* = 71, 72%), only 23% of the parents in these cases reported the use of illicit drugs. In cases with illicit drug detection, the rate of co-sleeping remained similar to the total co-sleeping rate (*n* = 46, 78%), however the number of infants placed in dangerous sleep positions was higher (*n* = 21, 38%). Less than half of the infants in this study (*n* = 41, 42%) were known to CPS based on the Department of Human Services response forms.

**Table 1:** 11 risk factors collected in 99 infant death cases

Risk Factor	Cases Present *n* (%)
Male Sex	62 (63%)
Co-sleeping	59 (78%)
Smoke exposure	52 (80%)
Parental unemployment	51 (81%)
*One parent employed	*26 (41%)
*Both parents unemployed	*25 (40%)
CPS Status	41 (42%)
No pacifier use	38 (62%)
Dangerous (non-supine) sleep position	25 (36%)
Reported illicit drug use	16 (25%)
Not breastfed	16 (23%)
Single/separated parents	15 (23%)
Not up to date with immunisations	9 (16%)

### Toxicology findings

Sixty different drugs were detected in hair amongst all cases. The total number of drugs detected in hair ranged from 1 to 13, with a median of 3 drugs detected in each case. Methylamphetamine was the most commonly detected drug (*n* = 57, 58%), followed by morphine (*n* = 35, 35%), codeine (*n* = 24, 24%) and cocaine (*n* = 22, 22%) (Table 2). Metabolites of certain drugs were also detected, most commonly amphetamine (*n* = 35, 35%). Polysubstance exposure was high (*n* = 62, 87%), especially in cases with illicit drug detection, where there was a median of 4 drugs per case. Of the 99 cases of drug detection in hair, 31 (31%) were complemented by drug detection in blood.

**Table 2:** Toxicology findings in 99 infant deaths with positive hair toxicology, with metabolites noted below each drug if detected

Drug Present	Cases Detected n(%)
Methylamphetamine	57 (58%)
*Amphetamine	*35 (35%)
Morphine	29 (29%)
Codeine	24 (24%)
*Codetection with morphine	*7 (7%)
Cocaine	22 (22%)
*Benzoylecgonine	*8 (8%)
*Other cocaine metabolites	*1 (1%)
Tramadol	22 (22%)
Delta-9-tetrahydrocannabinol	21 (21%)
Methadone	16 (16%)
*EDDP	* 11 (11%)

## Pathology findings

Almost half of the cases in this study were classified as unascertained (*n* = 49, 50%), with SIDS accounting for a further 29 (29%) deaths in this study. Of the unascertained cases, 40 (82%) occurred while the infant was asleep, 61% of which involved co-sleeping. Of the SIDS cases, 24 (83%) occurred in the context of co-sleeping. A full breakdown of the causes of death can be found in Table 3.

**Table 3:** Causes of death in 99 infant deaths with positive hair toxicology

Cause of Death	Cases Detected n(%)
Unascertained	49 (50%)
*During sleep	*40 (82%)
*While co-sleeping	*30 (61%)
SIDS	29 (29%)
*During sleep	*29 (100%)
*While co-sleeping	*24 (83%)
Natural Disease	12 (12%)
*During sleep	*5 (42%)
*While co-sleeping	*3 (25%)
Injury	7 (7%)
Other	2 (2%)

**Fig. 1:**
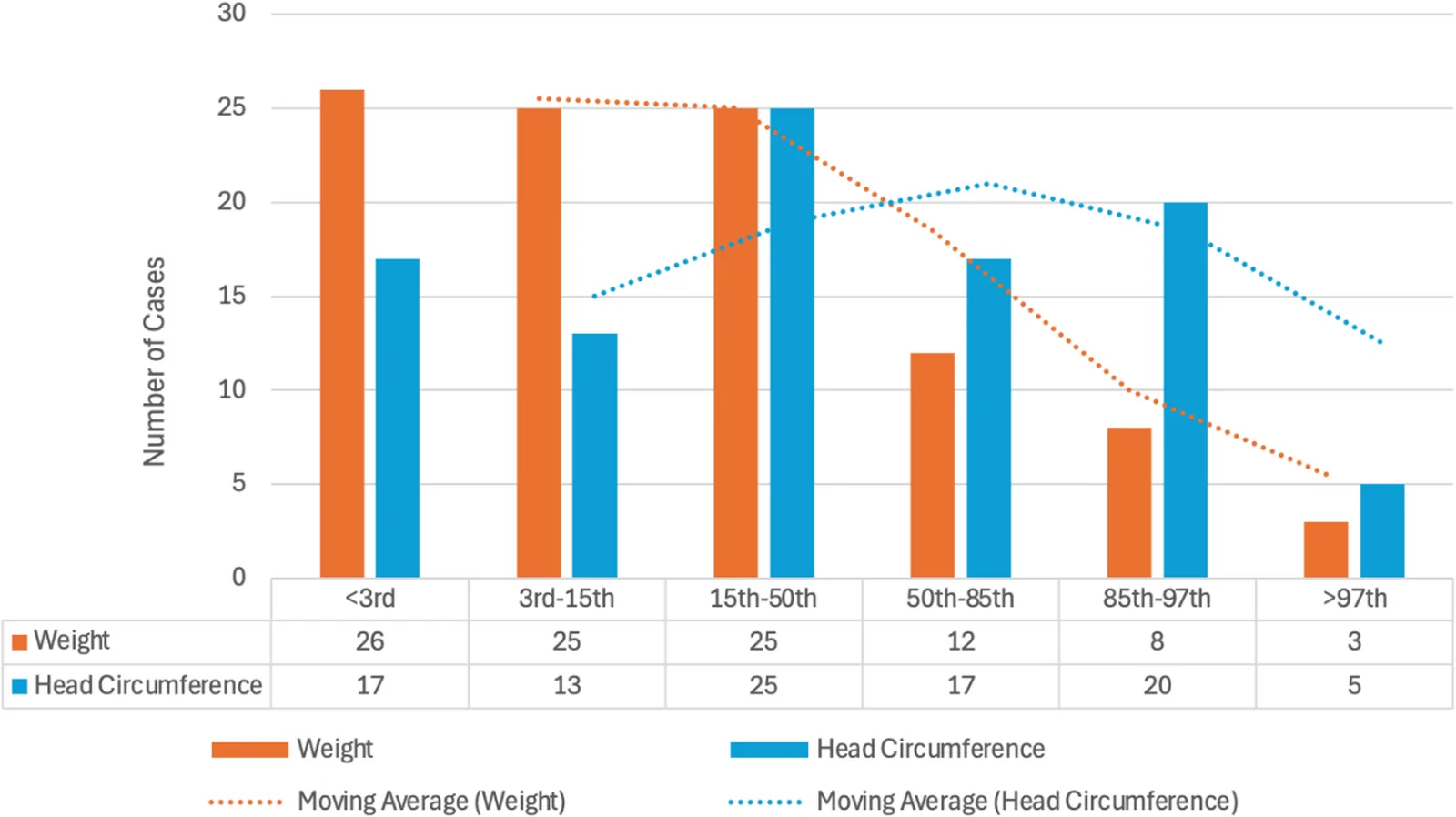
The body weights and head circumferences of the 99 infants across the WHO percentiles

Congenital abnormalities (*n* = 13, 13%), serious illness (*n* = 18, 18%) and serious neuropathology (*n* = 27, 27%) were commonly reported, with serious neuropathology most common. Congenital abnormalities were not associated with any specific drug/s (*p* = 0.845). However serious illness was significantly positively correlated with methylamphetamine detection (*p* = 0.002), while significant neuropathology findings were associated with morphine (*p* = 0.002) and codeine (*p* = 0.035) detection. The < 3rd percentile was most common for infant weight (*n* = 26, 26%) and the head circumference was most common in the 15th −50th (*n* = 25, 25%) (Fig. 1). Small head circumferences, at or below the 15th percentile, were significantly associated with methadone detection (*p* = 0.035). Methylamphetamine was not associated with infants at or below the 15th percentile for weight (*p* = 0.196), however amphetamine (*p* = 0.022) and morphine (*p* = 0.011) were.

## Discussion

Our study revealed a pattern of cumulative risk factors for SIDS among infants with positive hair toxicology, with every case demonstrating multiple co-existing risk factors, most notably co-sleeping and polysubstance exposure. The strong correlation between drug detection and adverse postmortem findings highlights the need to address environmental instability and substance exposure as modifiable contributors to SUDI risk in Australia. However, this study also noted inconsistencies in the classification of the deaths, more specifically the classification of SIDS versus ‘unascertained’, which may reflect a diagnostic shift previously described [28].

In all cases, at least one additional risk factor was identified alongside the presence of drugs, commonly prone or non-supine sleep positioning, and/or exposure to cigarette smoke. The majority of these infants died of SIDS or apparently during sleep with a negative autopsy, alongside the presence of these risk factors. This aligns with Filiano and Kinney’s *Triple Risk Model*, which suggests that SIDS cases often present with at least one risk factor, with an exponentially increased overall risk as the number of individual risk factors increases [8]. Previous studies reported at least one risk factor in 99% of cases, consistent with this study which identified risk factors in 100% of cases [10]. Co-sleeping was common, reported in over three quarters of cases. This finding diverges from previous Australian studies which suggest a notably lower prevalence of co-sleeping, reported at approximately 45% [29]. Given these results differ from other Australian studies, this may be an indication that the subgroup of SUDI cases with positive hair toxicology differs from the general SUDI population, which may be attributed to the chaotic home environment associated with parental drug use [13, 14]. It may also be associated with differing levels of parental self-reporting of risk factors [10].

Almost three quarters of cases tested positive for at least one illicit drug, most commonly methylamphetamine. Given that hair testing is not routinely performed in many jurisdictions, in combination with the sparsity of research in this area, it is unclear if illicit drug detection is common amongst SUDI cases more broadly. Hair toxicology is routinely performed in cases of infant death in Victoria, however this is not the case globally. Therefore, this study suggests that exposure to illicit drugs within the SUDI cohort may be a more serious and prevalent risk than is currently recognised. Our study reiterates previous findings that self-reporting bias can be an issue in this context, with 77% of cases not reporting the use of illicit drugs where they were indeed detected [30]. This highlights a major limitation of studies relying on parental self-reporting to determine drug use or exposure. Interestingly, parents who reported illicit drugs also reported a higher number of other risk factors. Most notably, while the co-sleeping rate for cases with illicit drug detection was the same as the total co-sleeping rate in this study (78%) the rate of infants being placed in dangerous sleeping positions was slightly higher in cases with illicit drug detection in hair compared to the rate for all drug detection (both illicit and non-illicit) in this study (38% and 36% respectively). Given that 86% of the infants in this study had siblings, it is possible that parents were less inclined to report factors they felt may reflect poorly on them, out of fear of losing their other children. The Victorian Government reports that 40% of child protective cases in Victoria involve parental drug use [31]. The guilt and shame associated with parental drug use in combination with the fear of losing their remaining children may present a barrier in honest reporting [32].

The most common cause of death in these cases was ‘unascertained’ (*n* = 49, 50%), followed by SIDS (*n* = 29, 29%). However, inconsistencies were noted in the classification of deaths (e.g. if the death was classified as unascertained vs. SIDS) for the cases in this study. A standard definition, known as the *San Diego definition*, was developed in 2004 and defined SIDS as “‘the sudden unexpected death of an infant < 1 year of age, with onset of the fatal episode apparently occurring during sleep, that remains unexplained after a thorough investigation, including performance of a complete autopsy and re-view of the circumstances of death and the clinical history” [7]. Though, due to the lack of pathognomonic findings for these cases, as highlighted in the above definition, they may also be classified as unascertained, potentially contributing to the variations in classification. These inconsistencies across the classification of infant deaths is a finding common to other studies [28, 33]. Research suggests that this may be due to the *San Diego definition* not being uniformly applied [7]. A recent study evaluating 264 papers on SIDS between 2019 and 2021 reported that only 35% quoted or referenced a standard definition [33]. This was a significant decrease from the 68% of studies correctly utilising this definition a decade earlier [34]. In a review of the current classification systems, Byard et al. [35], highlighted major inconsistencies in the classifications of infant death. They suggested that this may be a result of ongoing ambiguities in the definition in combination with the lack of pathognomonic markers often noted in SIDS investigations, contributing to the inconsistent application.

Of the five markers collected for pathology results (serious illness, congenital abnormalities, serious neuropathology, head circumference and body weight), all were significantly correlated with one or more of the detected drugs, aside from congenital abnormalities. Serious neuropathology findings were correlated with morphine and codeine detection in hair. While they can both be primary drugs, they can also be metabolites of heroin [36]. In blood, a ratio of morphine to codeine that is 2:1 or greater is suggestive of heroin use, however this ratio does not align with hair toxicology results, which are more challenging to interpret [37]. This is because drug incorporation into hair is influenced by factors such as growth rate, cosmetic treatments, and environmental contamination, which do not directly reflect recent dosage or timing of use. Therefore, the absence of heroin specific metabolites (e.g. 6-MAM) does not exclude the possibility of heroin [38]. This research highlighted that serious illness is significantly correlated with exposure to methylamphetamine. This is consistent with a study exploring prenatal methylamphetamine exposure and short-term infant health outcomes, which reported that exposed infants were more likely to be admitted to the Neonatal Intensive Care Unit [39].

Additionally, morphine and amphetamine (likely derived from methylamphetamine) exposure were associated with lower body weights. This is consistent with previous studies that found infants exposed to methylamphetamine in utero were significantly more likely to show growth restriction, even when born at full term [40]. The high rate of codetection of methylamphetamine and amphetamine in our study suggests that methylamphetamine was the primary drug, indicative of higher levels of exposure than methylamphetamine detection alone [37]. This may represent a dose-dependent relationship between methylamphetamine exposure and weight; a finding supported by Wright et al. [41], who reported that the amount of prenatal exposure to methylamphetamine impacted birth outcomes, and that reducing maternal methylamphetamine use at any point during pregnancy improved the birth outcome for these infants. They found that infants exposed to methylamphetamine throughout the entire pregnancy were at higher risk for adverse outcomes. This was consistent with the findings of our study where high levels of exposure to methylamphetamine, determined through the codetection of amphetamine, were commonly associated with adverse postmortem findings. This may be reflective of higher methylamphetamine use in the subgroup of SUDI with positive hair toxicology, or the higher toxicity risk for infants with exposure to this drug. Similarly, methadone was associated with smaller head circumferences, consistent with previous findings [42]. These results not only suggest that exposure to drugs, particularly illicit drugs, in cases of SUDI and SIDS may play a significant role in increasing risk, but also that this can be correlated to many of the specific pathology findings we explored. Through the correlation of drug exposure to pathological findings, we may be in a better position to characterise and understand these cases of infant death.

It is important to note that hospital records were not available for this study, therefore it was not always possible to determine whether some drugs had been clinically administered. The time delay for drug absorption into hair required for positive detections suggests that these results may reflect prior exposure, however drugs can be incorporated into hair within days. Therefore the route of administration cannot be said with certainty for drugs commonly administered in a hospital setting, including morphine and codeine. While this study explored drug-pathology associations individually, it did not account for drug interactions or the implications of polysubstance use. In doing so, valuable information or relationships may have been missed. Additionally, drug testing in hair is continuously advancing, allowing more sensitivity in drug detection. However, drugs present in concentrations below the limits of reporting would not be documented in these cases [43].

Our findings suggest that cases of SUDI with positive hair toxicology are complex and require a nuanced understanding of the multiple factors which may contribute to these deaths. The high rate of illicit drug detection among SUDI cases indicates that this may be more common than is currently recognised. The high rate of polysubstance use among SUDI cases, in conjunction with a high presence of multiple simultaneous risk factors for SIDS and potential dose-dependent relationships between drugs and pathology outcomes highlights opportunities for targeted harm prevention strategies among parents who use drugs. This is especially important given the high number of infant deaths which remain unascertained following investigation and the recent plateau in Australian SUDI rates [4, 6]. While this can be partially attributed to the inconsistencies surrounding SIDS diagnoses, it largely underscores the lack of research and understanding in this area.
